# A muscle synergies-based controller to drive a powered upper-limb exoskeleton in reaching tasks

**DOI:** 10.1017/wtc.2024.16

**Published:** 2024-11-15

**Authors:** Michele Francesco Penna, Luca Giordano, Stefano Tortora, Davide Astarita, Lorenzo Amato, Filippo Dell’Agnello, Emanuele Menegatti, Emanuele Gruppioni, Nicola Vitiello, Simona Crea, Emilio Trigili

**Affiliations:** 1 The BioRobotics Institute, Scuola Superiore Sant’Anna, Pontedera, Italy; 2 Department of Excellence in Robotics & AI, Scuola Superiore Sant’Anna, Pisa, Italy; 3 Department of Information Engineering, University of Padova, Padova, Italy; 4 Padova Neuroscience Center, University of Padova, Padova, Italy; 5 Centro Protesi Inail di Vigorso di Budrio, Bologna, Italy

**Keywords:** Exoskeletons, Intention decoding, electromyography, wearable robotics

## Abstract

This work introduces a real-time intention decoding algorithm grounded in muscle synergies (Syn-ID). The algorithm detects the electromyographic (EMG) onset and infers the direction of the movement during reaching tasks to control a powered shoulder–elbow exoskeleton. Features related to muscle synergies are used in a Gaussian Mixture Model and probability accumulation-based logic to infer the user’s movement direction. The performance of the algorithm was verified by a feasibility study including eight healthy participants. The experiments comprised a transparent session, during which the exoskeleton did not provide any assistance, and an assistive session in which the Syn-ID strategy was employed. Participants were asked to reach eight targets equally spaced on a circumference of 25 cm radius (adjusted chance level: 18.1%). The results showed an average accuracy of 48.7% after 0.6 s from the EMG onset. Most of the confusion of the estimate was found along directions adjacent to the actual one (type 1 error: 33.4%). Effects of the assistance were observed in a statistically significant reduction in the activation of Posterior Deltoid and Triceps Brachii. The final positions of the movements during the assistive session were on average 1.42 cm far from the expected ones, both when the directions were estimated correctly and when type 1 errors occurred. Therefore, combining accurate estimates with type 1 errors, we computed a modified accuracy of 82.10±6.34%. Results were benchmarked with respect to a purely kinematics-based approach. The Syn-ID showed better performance in the first portion of the movement (0.14 s after EMG onset).

## 1. Introduction

Following a stroke, intensive motor training can improve the recovery of sensorimotor functionalities (Langhorne et al. 2011). Robotic rehabilitation allows patients to actively participate during the exercises (Lo et al. 2010; Skidmore et al. 2010), with adaptive and personalized level of support, fostering the engagement of the patients and enhancing the overall rehabilitation outcome. Additionally, robotic devices such as powered exoskeletons (Pan et al. 2022; Zimmermann et al. 2023) can be useful to promote task-specific training, which is typically correlated with a superior improvement of the upper-limb motor functionalities with respect to the usual care (Klamroth-Marganska et al. 2014; Winstein et al. 2016).

To provide a natural and intuitive experience and promote neuroplasticity, exoskeletons must be controlled according and synchronously to the user’s motor intention, amplifying spontaneous residual movements (Balasubramanian et al. 2018; Li et al. 2018). To do so, intention decoding algorithms (IDAs) can rely on the kinematic or biosignal information acquired and processed by the exoskeleton’s sensory and control systems (Tucker et al. 2015).

Kinematic signals can be used as inputs of IDAs to provide human augmentation during rhythmic or discrete tasks (Lanotte et al. 2021; Penna et al. 2023; Ronsse et al. 2011; Sanz-Morère et al. 2021). However, since these approaches rely on the observation of the user’s limb movement, they are not suitable for those subjects suffering from severe motor disorders, who would benefit from an external aid to initiate the movement. This problem can be addressed by monitoring the nervous system activities associated with the control of voluntary movements. Such an approach would provide additional time to the robot’s controller, leveraging the temporal gap between the generation of electrical signals by the nervous system and the subsequent generation of force in the muscles (and consequent limb movement), commonly referred to as electromechanical delay (Cavanagh and Komi 1979). Surface electromyography (EMG) can sense the peripheral nerve signals using a noninvasive technique that entails placing electrodes on the skin over the area of the muscle of interest. Despite inherent limitations related to noise caused by crosstalk, variability of the skin impedance, for example, due to sweat, and movement artifacts (Farina et al. 2014), a great evidence has been gathered over the years demonstrating the effectiveness of using EMG signals as inputs of IDAs. EMG signals can be used to extract features both in the time and frequency domains (Bi et al. 2019; Englehart et al. 1999), which can serve as inputs to infer human motor intentions using model-based and/or machine learning techniques (Kiguchi and Hayashi 2012; Lotti et al. 2020; Novak et al. 2013; Trigili et al. 2019).

EMG signals can be processed to extract muscle synergies, namely coordinated muscle activity patterns. According to this theory, the central nervous system controls the execution of movements through a reduced set of synergistic and coordinated muscle groups (d’Avella et al. 2003). Synergies have several characteristics that make them good candidates for being input signals of IDAs. First, they are robust to high-variance changes typical of EMG acquisitions (Ison and Artemiadis 2014). Second, they allow to model EMG activations in a lower-dimensional features domain, by reducing the number of controlled variables (Berniker et al. 2009). Finally, upper-limb muscle synergies were shown to be robust to differences in motor performance and differences in cerebral lesion sizes and locations between poststroke patients (Cheung et al. 2009). Grounded on these considerations, several attempts were made to employ muscle synergies to classify single joint movements of the shoulder, the elbow (Antuvan et al. 2016), and of the forearm (Tse et al. 2023), as well as multi-joint reaching tasks (Israely et al. 2018). However, to date, very limited attempts have been made to incorporate muscle synergies into exoskeleton controllers, with a few exceptions limited to the lower limbs, showing promising results (Alibeji et al. 2018; Wei et al. 2020).

To overcome this knowledge gap, this work introduces and verifies a novel IDA exploiting muscle synergies, named Syn-ID algorithm. Starting from the observation of the subject’s EMG signals, the algorithm is able to (i) detect the muscle activation onset, (ii) infer the intended movement direction, and (iii) control an upper-limb exoskeleton to guide the subject’s hand toward eight different directions. The proposed approach was tested online with a shoulder–elbow exoskeleton (NEUROExos Shoulder-elbow Module- 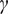 , NESM- 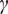 ) for the use case of planar reaching tasks. The remainder of the paper is organized as follows: Section 2 presents the NESM- 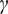 exoskeleton, the EMG acquisition system, the Syn-ID algorithm, and the design of the verification study. The results of the experiments are presented in Section 4; Section 5 discusses the results and draws the conclusions.

## 2. Materials and methods

### 2.1. NESM-γ upper-limb exoskeleton

**Figure 1.**
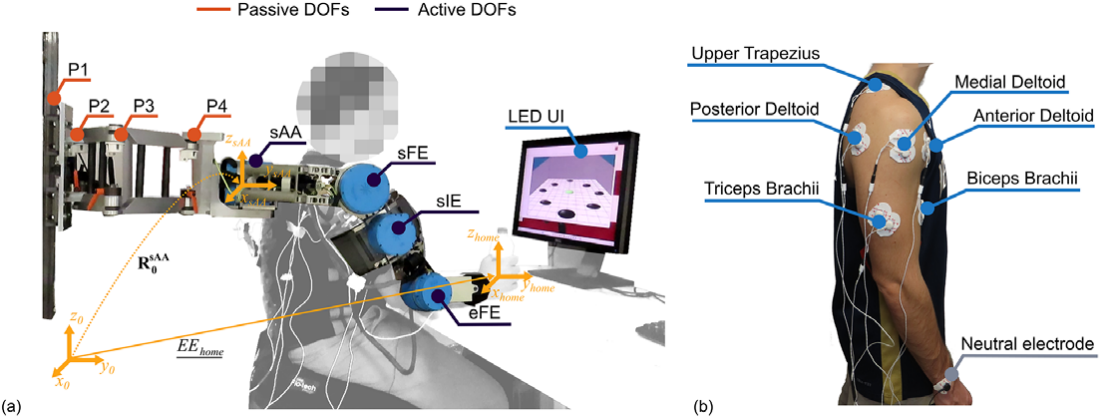
Experimental setup comprising (a) the NESM-γ exoskeleton with the user interface (UI) and (b) the EMG target muscles.

The NESM- 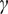 is a shoulder–elbow exoskeleton designed for neurorehabilitation applications, developed by the Wearable Robotics Lab (The BioRobotics Institute, Scuola Superiore Sant’Anna; Pan et al. 2022). The exoskeleton (Figure 1(a)) is equipped with four active degrees of freedom (DOFs), allowing shoulder abduction/adduction (sAA), flexion/extension (sFE), internal external rotation (sIE), and elbow flexion extension (eFE). Each of the active DOFs has a reactive-force series elastic actuation architecture with custom torsional springs and embeds two absolute encoders measuring the joint position and torque (the latter measured via the spring deformation). The exoskeleton is mounted on a support column with lockable wheels. The kinematic chain of active DOFs is connected to the support column by four passive DOFs (P1–4). This passive chain accounts for scapulohumeral rhythm and trunk rotation, and it has self-alignment purposes (Pan et al. 2023).

The control system of NESM- 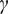 runs on two processors: (i) a field-programmable gate array (FPGA) processor, and (ii) a 667-MHz dual-core ARM processor running a National Instruments (NI, Austin, Texas, USA) RT operating system. The FPGA processor runs a low-level control layer at 1 kHz, which is responsible for the reading of the robot’s sensors and the execution of the closed-loop torque regulators, which translate the error between the reference and measured torque into a motor command for the current servoamplifiers driving the brushless DC motors. The digital input/output ports of the FPGA processor are configured to execute synchronous serial interface (SSI) and serial peripheral interface (SPI) communication protocols, facilitating the interaction of the robot with external data acquisition systems, for example, EMG acquisition systems. The ARM RT processor implements a control loop at a frequency of 100 Hz. This loop is responsible for the execution of the IDAs and the generation of the reference joint torques for each joint. These reference torques are computed as the sum of the desired torques resulting from the IDAs with a feed-forward gravity compensation term. This term compensates for the gravitational torques due to the exoskeleton’s weight and it is dependent from the exoskeleton’s pose. Using this control scheme, validated in (Pan et al. 2022), the robot can either be “transparent” to the user’s spontaneous movements (*transparent mode*, when the desired torques are set to 0 Nm) or selectively provide joint torques to assist the user’s movements, for example, creating a convergent force field toward reference attraction points (*assistive mode*).

### 2.2. EMG acquisition system

We recorded EMG signals from six upper-limb muscles (Figure 1(b)), namely (i) biceps brachii, (ii) triceps brachii (long head), (iii) anterior deltoid, (iv) medial deltoid, (v) posterior deltoid, (vi) upper trapezius. The signals were recorded by an EMG acquisition system composed by the SeWi electromyograph (OTBioelettronica, Torino, Italy) and a dedicated electronic board (SbRIO-9561, NI, Austin, TX, USA). Raw EMG signals are processed at 1 kHZ, implementing a band-pass filter between 20 and 400 Hz, a notch-filter at 50 Hz, a rectification, and, finally, a low-pass filter at 4 Hz. The processed signals, that is, the linear envelopes, are used by the electronic board to run the Syn-ID high-level control algorithm at 100 Hz. Finally, the estimate of the movement direction, that is, the output of the Syn-ID control algorithm is transmitted to the NESM- 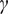 electronic board, via an SSI communication protocol.

### 2.3. The Syn-ID control algorithm

**Figure 2.**
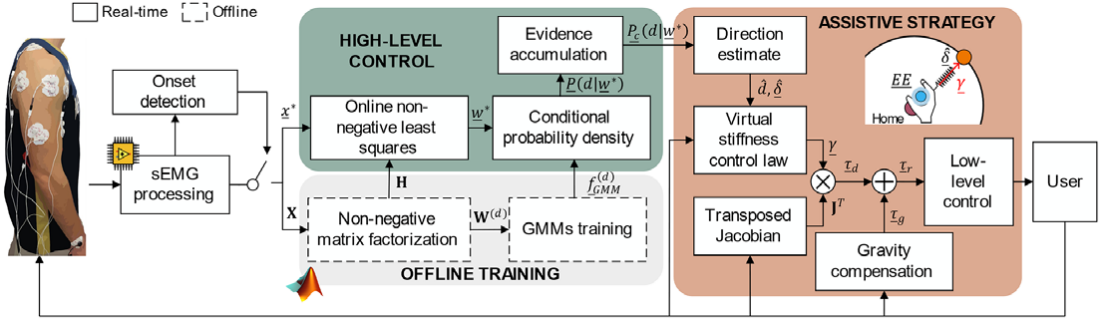
Block diagram of the algorithm. When the onset of the EMG activation is detected, the signals are used to extract the activation coefficients of the muscle synergies. The activation coefficients are used as input to a Gaussian Mixture Model-based strategy, whose outputs allow inferring the direction of the reaching movement. Finally, the exoskeleton is actuated with an impedance control-based strategy to guide the hand of the user toward the inferred direction. In this figure, the dependency of the variables on the program iteration 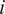 has been eliminated to enhance readability.

The proposed approach grounds on the following steps, performed in RT: (i) the system acquires EMG signals from upper-limb superficial muscles and extract informative features on muscle synergies, (ii) after the onset of the reaching movement is detected, a Gaussian Mixture Model (GMM) estimates the direction of the movement based on the above features, and (iii) the control system of the exoskeleton generates assistive torque profiles at the different joints to guide the user’s hand toward the desired target. Before running the algorithm, an offline procedure is implemented to extract the muscle synergies and train the GMMs. The block diagram of the algorithm is shown in Figure 2.

In the remainder of the article, the following mathematical notation conventions will be employed: matrices will be denoted in bold (e.g., 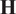 ), vectors will be represented as underlined (e.g., 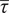 ), and scalars will maintain standard notation.

#### 2.3.1. Offline muscle synergies computation and GMMs training

For the offline muscle synergies computation and GMMs training, first, EMG signals were recorded while users performed reaching movements toward 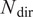 different directions, wearing the NESM- 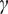 exoskeleton in transparent mode. Then, the procedures comprised the following steps:

1. *Non-negative matrix factorization.* The segmented envelopes were used as input to the non-negative matrix factorization (NMF) algorithm, which was implemented as described in Lee and Seung (2000), and hereafter briefly recapped. Given the matrix of envelopes 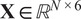 , where 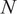 is the length of the acquisition, the NMF iteratively searches the matrices 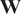 and 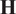 that minimize the root-mean-squared residual between 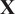 and 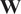 ⋅ 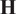 . The matrix 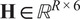 represents the synergies matrix, which accounts for the contribution of each muscle to the synergy modules (with 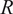 representing the number of muscle synergies to consider, that was set to four (Israely et al. 2018)). The matrix 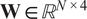 represents the activation coefficients of each muscle synergy throughout the acquisition time.
2. *Labeling.* Training activation coefficients, contained in matrix 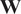 , were labeled with respect to their directions of movements, 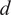 , considering the 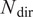 possible directions.
3. *GMMs training.* The activation coefficients associated with each movement direction were modeled as a weighted sum of three Gaussian components, resulting in a set of 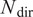 GMMs. Thus, the probability density function (PDF) of the GMM associated with the 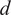 -th direction was computed as:(1)

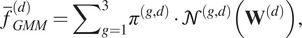

where 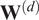 contained the activation coefficients of the movements toward the direction 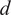 , and 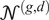 was the PDF the 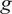 -th Gaussian component, and 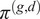 were the mixing coefficients weighing the PDF and satisfying 0 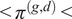 1 and 
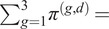
 1. The GMMs parameters were first initialized through a 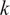 -means algorithm, with 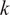 equal to three, and then estimated iteratively through an expectation-maximization algorithm.

#### 2.3.2. Onset detection algorithm

The onset of the reaching movements was recognized using a real-time EMG-based segmentation algorithm. The onset detection represented an extension of the threshold-based method described in Solnik et al. (2010), modified to accommodate the concurrent activation of multiple muscles of our case study. The algorithm computed the sum of the EMG envelopes of the six upper-limb muscles. A threshold 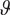 was then computed as:(2)

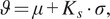

where 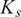 is a tunable gain, 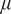 is the mean, and 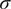 is the standard deviation of the sum signal in a moving window of 150 ms. When the sum signal was greater than the threshold 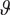 , the onset of the EMG activation was detected.

#### 2.3.3. Real-time high-level control

The real-time high-level controller computed an estimate of the reaching movement direction ( 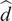 ) observing an initial portion of the EMG activation patterns, from the detected movement onset 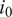 , to a given time instant 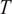 , called *accumulation time.* After the onset 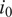 , at each program iteration 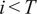 , the algorithm performs the following steps:

1. *Online non-negative least squares.* Given the observed linear envelopes 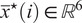 , the associated synergy activation coefficients 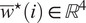 were estimated solving a non-negative least squares constraint problem:(3)

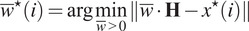

2. *Conditional probability logics.* The conditional probability density of each movement direction 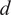 , given the estimated activation coefficients 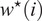 and the related PDF of the GMM obtained during the offline training 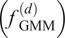 , is computed from the GMM as:(4)

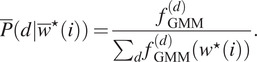

  The vector of conditional probability density 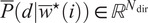 , was then normalized so that 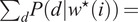 1.
3. *Evidence accumulation.* An evidence accumulation approach was used to increase the confidence over the most probable direction; a cumulative conditional probability 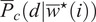 was computed for each direction as:(5)

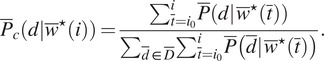

Again, the cumulative probabilities were normalized so that 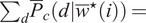 1.
4. *Direction estimate.* The movement direction was estimated as the one with the highest cumulative probability, as:(6)

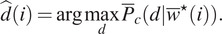

The estimate of the target 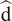 evolved according to (6) until the accumulation time 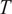 . Once the accumulation time was elapsed, the estimate was kept fixed as its value at 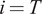 .

#### 2.3.4. Assistive strategy

Along the movement, the NESM- 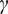 exoskeleton was controlled to assist the reaching movement toward a target position ( 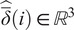 ) associated with the estimated direction 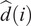 :

1. *Virtual stiffness.* A virtual stiffness control law was implemented to guide the movement of the user’s hand toward the estimated target position. At each iteration 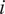 , the position of the user’s hand, 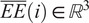 in the global frame was computed through direct kinematics (Pan et al. 2022). 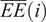 and 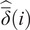 were reported in the sAA frame (i.e., the first joint of the active kinematic chain) to account only for the active chain for the generation of the assistive force.
  A virtual stiffness 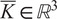 was used to compute the virtual force 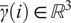 , which was the force necessary to direct the hand 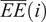 toward the estimated target position, that is, the attraction point:(7)

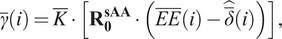

where 
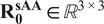
 was the rotational matrix from the NESM- 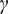 global frame to the sAA joint (Figure 1(a)), and it was a function of the passive degrees of freedom.
2. *Reference torques computation.* Finally, the joint reference torques 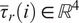 were computed as:(8)

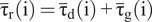

where 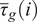 were feed-forward, pose-dependent gravity compensation torques, and were a function of 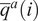 (Pan et al. 2022), while 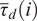 were the desired assistive torques, computed applying the statics equation of the active kinematic chain of the robot:(9)

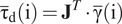




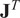
 was the transposed Jacobian matrix of the active kinematic chain of the NESM- 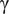 .

### 2.4. Study design

In order to verify the performance of the Syn-ID control algorithm, we conducted a pilot study with eight healthy participants (S1–S8, 5M/3F, height 173 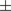 8.8 cm, weight 71 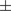 13 kg). The experimental procedures were approved by the Ethics Committee of Scuola Superiore Sant’Anna, Pisa, Italy (approval no. 34/2021) and conducted in accordance with the principles stated in the Declaration of Helsinki. The participants signed written informed consent to participate in the study.

Upon recruitment, electrodes for EMG were attached to the six target upper-limb muscles (Figure 1(b)). The participant was asked to perform three repetitions of 5-second maximum voluntary isometric contractions (MVC) for each muscle (Burden 2010). The MVC data were only used for offline data analysis, while the Syn-ID algorithm used the non-normalized linear envelopes. The protocol included two sessions, namely a *transparent* and an *assistive* session. During the transparent session, the subject was asked to perform eight reaching movements toward eight different targets marked on a table, while the NESM- 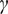 was operated in transparent mode. The targets were equally spaced on a 25 cm diameter circumference on the four cardinal (north (N), east (E), south (S), and west (W)) and the four ordinal directions (north-west (NW), south-west (SW), south-east (SE), and north-east (NE)), so that the angular distance between adjacent targets was 45 deg. All the reaching movements started with the subject’s hand in a home position, which was marked on the table at the centre of the circumference and aligned along the sagittal plane with the subject’s shoulder joint. A representation of a subject with the hand in home position is shown in Figure 1. To guide the execution of the movements, the target positions were shown to the user via LEDs on a graphical user interface (LED UI; Figure 1(a)). The LEDs were programmed so that each one would turn on for 5 s, in a randomized sequence. Once the target LED turned off, the subject was asked to return to the home position, which was also indicated by a central LED. Throughout the movement execution, the subject was instructed to focus on the LED UI and maintain a consistent pose through the experiment while in the home position. Every 5 minutes, short breaks were allowed to reduce the effects of muscle fatigue. The processed EMG signals, segmented between 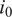 and 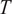 , that is, the onset instant and the accumulation time, were used to compute the muscle synergies and train the GMMs classifiers, as explained in Section 2.3.1. The accumulation time 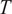 was fixed to 0.6 s. The transparent session lasted approximately 15 minutes.

During the assistive session, the subject performed the reaching movements while the exoskeleton was operated in assistive mode, implementing the Syn-ID algorithm. The exoskeleton was programmed to assist the reaching movement and support the arm in position after the task, until the LED turned off. At the beginning of the session, a quick calibration procedure was performed to identify the position of the targets in the workspace ( 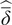 ): with the robot in transparent mode, the subject was asked to keep a stable position on the home target and the 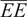 value was acquired. Then, the 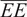 positions on the other targets were obtained via geometrical considerations. After the calibration procedure, participants were instructed to perform some movements toward the eight targets in assistive mode, while the experimenter fine-tuned the value of 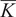 , starting from a reference value ([0.01, 0.03, 0.01] N/m), until the participant felt comfortable with the level of assistance. Finally, a total of 20 reaching movements toward each target were performed in the assistive mode with the tuned stiffnesses. For these movements, the accumulation time 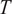 was fixed to 0.6 s. Every 5 min, short breaks were allowed to avoid muscle fatigue. The assistive session lasted approximately 30 minutes. A video representing the execution of the experiment from a representative subject can be found in Supplementary materials.

### 2.5. Data analysis

The data collected in the experimental sessions were analyzed offline using custom MATLAB routines. The performance of the Syn-ID algorithm was assessed computing the following key performance indicators.

1. *Accuracy.* Given the number of true positives 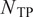 (i.e., movement directions correctly recognized at 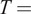 0.6 s) and 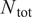 the total number of movements, the accuracy of the algorithm was computed as:(10)

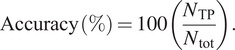

2. *Error types.* Considering the geometry of the problem, the possible estimate errors were categorized into four different error types, according to the location of the misclassified target relative to the expected one:
  - type 1 error: misclassification to a direction adjacent to the expected one (e.g., N vs. NW);
  - type 2 error: misclassification to a direction perpendicular to the expected one (e.g., N vs. W);
  - type 3 error: misclassification to a direction near-opposite to the expected one (e.g., N vs. SW); and
  - type 4 error: misclassification to a direction opposite to the expected one (e.g., N vs. S).
  - For each type, an error percentage was computed as in equation (10).
3. *Modified accuracy.* A modified accuracy value was computed as the sum of accuracy and type 1 errors.
4. *Movement repeatability.* The repeatability of the movements was quantified computing two indicators in the home reference frame (EE_home_, *x*_home_, *y*_home_, *z*_home_; Figure 1(a)), that is, the reference system parallel to the global frame and centered in the home position of the workspace, using polar coordinates. The final position of the hand at the end of the assisted movement and a reference centroid for each target, computed as the average final hand position of the transparent movements toward that target, were transformed into polar coordinates using:(11)

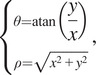

resulting in 
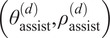
 and 
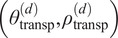
 , respectively. Then, for each movement, we computed two error metrics, expressing the discrepancies between movements in assisted and transparent mode, in terms of angular value and hand advancement with respect to the targets 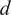 :(12)

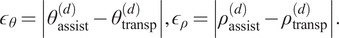

5. *Muscle activations.* The effects of the assistance were evaluated computing the integral of the EMG signals (iEMG) for the transparent and assistive sessions. Before computing the iEMG, the signals were normalized in time between 0% and 100% and in amplitude with the MVC of each muscle.
6. *Manipulability indexes.* The manipulability indexes for the movements of the transparent and assistive sessions toward each target was computed as:(13)

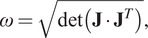

where 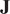 is the Jacobian matrix of the active kinematic chain of NESM- 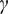 and 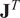 is its transpose. The manipulability index is bounded between 0 and 1 and it depends on the exoskeleton’s configuration. When 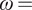 0, the exoskeleton is in a singular configuration, where small end-effector velocities cause large joint velocities; thus, higher values of 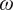 (i.e., closer to 1) are preferred. For each movement, a single manipulability index was computed as the mean of the 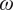 observed from the initial position toward the target. Before computing 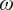 , movements were normalized in time between 0% and 100%.

Finally, the performance of the muscle synergies-based ID algorithm in estimating the reaching movement direction was benchmarked offline with respect to a state-of-the-art kinematics-based ID algorithm. Given the end-effector position 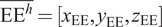 in the home reference frame, the angular polar coordinate 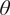 was computed at each time instant 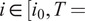 0.6 s 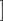 as in (11).

Then, the direction 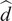 was estimated as the one minimizing:(14)

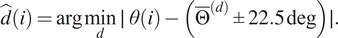



Where 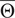 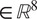 contains the angular polar coordinate of the actual targets, spaced at intervals of 45 deg. For example, a movement was classified toward the N direction ( 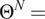 90 deg) if 67.5 < 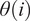 < 112.5 deg.

### 2.6. Statistical analysis

Statistical significance changes in both iEMG values and manipulability coefficients across the transparent and assisted sessions were investigated. Moreover, the possible significant differences in the performance of the implemented muscle synergy-based ID algorithm and the offline benchmark were evaluated. Outlier detection was conducted using Tukey’s method. The indicators were tested for normality and homoscedasticity using the Kolmogorov–Smirnov test and Levene’s test, respectively. None of the tested indicators resulted normally distributed. Therefore, the following tests were performed:

1. A bidirectional Wilcoxon signed rank test was used to check for possible significant differences between transparent and assistive sessions, for iEMG values, and manipulability indexes.
2. A Friedman’s test was used to check for potential direction-dependent significant differences in the manipulability indices. For comparisons yielding significant differences among the tested conditions, a post-hoc analysis was performed using the Tukey’s honestly significant difference correction.
3. Two one-tailed Wilcoxon signed rank tests were used to assess possible statistically significant differences between the muscle synergy-based ID algorithm and the offline benchmark.

All statistical analyses were performed in MATLAB with a significance level 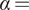 0.05.

## 3 Results

Three movements of the assistive sessions (0.24% of the total) were excluded from the data analysis due to onset detection errors, that is, the onset was not timely detected for estimating the movement direction; moreover, a specific movement performed by S8 (assisted movement toward SE) was excluded from this analysis, due to signal noise on the Biceps Brachii reading, likely caused by a sudden movement of the subject against the NESM- 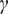 upper-arm interface.

**Figure 3.**
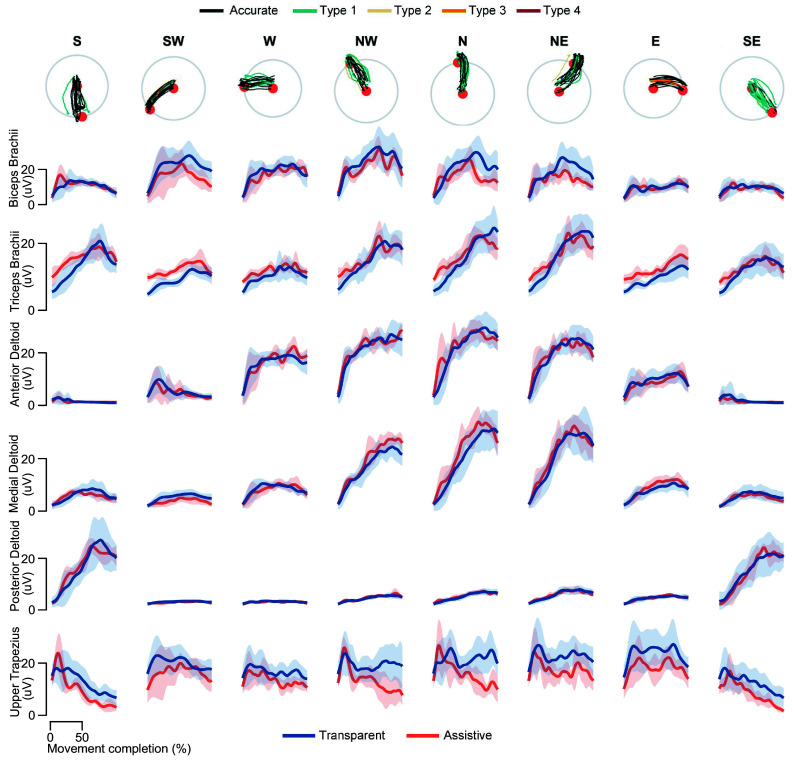
Data from a representative subject, grouped by movement direction. The first row shows the user hand trajectories from during the assistive session, where the orange dots represent the average initial and final positions during transparent session. The remainder rows of the figure show the mean 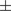 standard deviation EMG linear envelopes in the transparent and assistive sessions. Muscles activities are normalized in duration considering as onset the instant detected by the onset detection algorithm and as end of the movement the instant identified by a noncausal state-of-the-art algorithm using kinematic activities.

Figure 3 shows the hand trajectories of a representative subject (S4) toward the eight targets during the assistive session, along with the mean 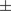 standard deviation EMG activations of both transparent and assistive sessions. Trajectories were normalized in duration considering as 0% the instant detected by the EMG onset detection algorithm, and as 100% the instant detected by a state-of-the-art noncausal kinematics-based algorithm, that is, the time instant on which the 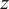 coordinate of 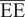 , decreases to a value equal to 20% of its peak value during the movement, similarly to (Trigili et al. 2019). The 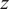 coordinate was chosen because it had a bell-shaped profile for all targets. Most of the hand trajectories reached the target end position, showing consistent patterns regardless of the error type. The direction that was estimated more accurately for S4 was SW (85% of accuracy), while the least accurate was SE (20% of accuracy), which was confused with S in 70% of instances. The final hand positions during movements toward adjacent targets were notably close one to each other. Among all, the centroids of N and NW targets were the closest ones (8.35 cm apart in the 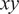 plane), while the centroids of S and SE were 14.92 cm apart in the 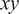 plane. Notably, the Syn-ID algorithm confused N with NW 35% of cases, and NW with N the 21% of instances. Instead, S was confused with SE 28% of cases, while SE was confused with S 30% of instances. Consistent patterns could be observed in the EMG activations between transparent and assistive sessions. The action of the arm and forearm flexor muscles (i.e., Anterior Deltoid and Biceps Brachii) peaked for movements toward N, NE, and NW, while the extensor muscles (i.e., Posterior Deltoid and Triceps Brachii) activated mostly for movements toward S, SE and SW. Notably, the Posterior Deltoid exhibited the highest level of activation among all muscles for movements toward the S and SE directions. Finally, for both transparent and assistive movements toward all the directions, the Biceps Brachii kept a bell-shaped pattern of activation, with a peak around 50% from the EMG onset.

**Figure 4.**
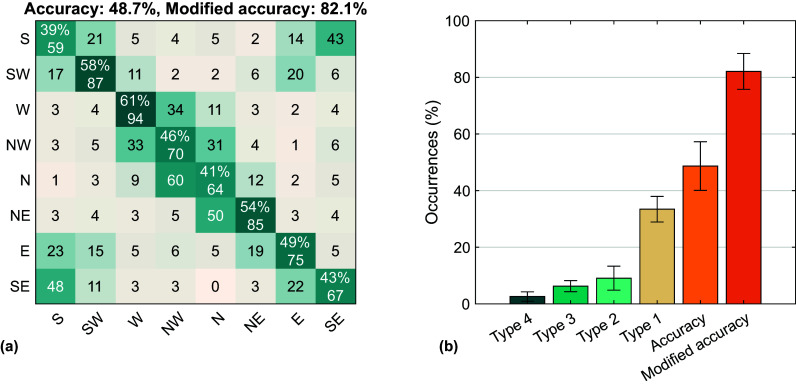
Overall performance of the Syn-ID algorithm. (a) Confusion matrix of the direction estimate; (b) accuracies and estimate errors, averaged on subjects. Modified accuracy is computed as the sum of Accuracy and Type 1.

The confusion matrix for the ID algorithm based on muscle synergies is reported in Figure 4(a), resulting in an average accuracy of 48.6% and modified accuracy of 82.1%, at 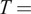 0.6 s. The directions that, on average, were estimated more accurately were W and SW (61% and 58%, respectively), while those that have been estimated less accurately were S and N (39% and 41%, respectively). The directions with the highest modified accuracy were NE (88%) and NW (87%), while those with the lowest modified accuracies were E (65%) and SW (76%). Figure 4(b) shows the accuracies and the estimate errors at 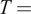 0.6 s, averaged on the participants. Most of the tested movements were estimated accurately (48.6 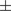 8.5%), while most of the confusion was detected along adjacent directions, as reflected by the errors of type 1 being the most frequent errors (33.4 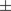 4.5%). Modified accuracy was 82.1 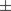 6.3%. Most severe errors, that is, type 4, were also the least frequent ones (2.5 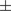 1.6%).

**Figure 5.**
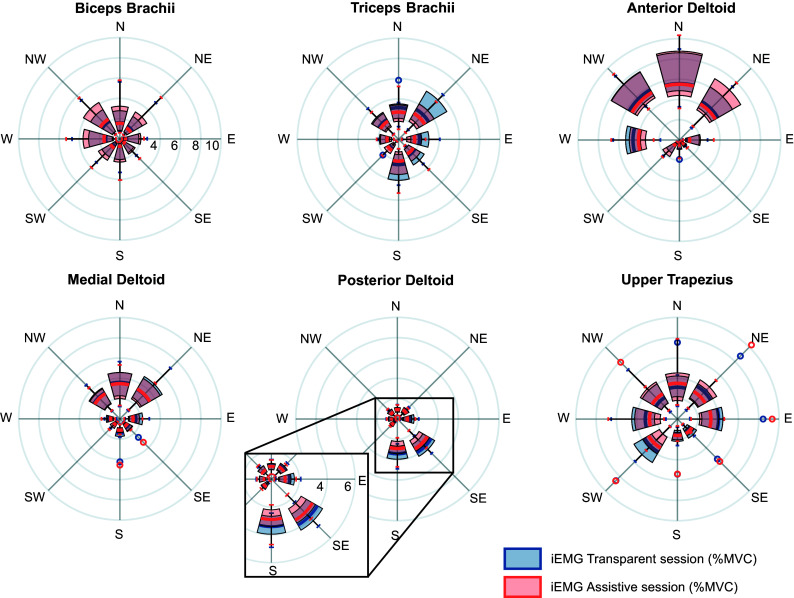
Boxplots of the distribution of the integral EMG (iEMG) values aggregated on the eight participants. For each muscle, the iEMG distributions are shown orienting the boxplot according to the movement direction.

The iEMG values aggregated on participants are reported in Figure 5. Aggregating data over directions, four out of six muscles reduced their median iEMG between the transparent and assistive sessions, with the exception of Biceps Brachii and Anterior Deltoid (+6.21% and +8.45%, respectively, but without statistical significance). Triceps Brachii, Medial Deltoid, Posterior Deltoid, and Upper Trapezius exhibited a reduction in the assistive session (−5.39%, −4.75% −6.10%, −7.17%, respectively). The difference of the Triceps Brachii and of the Posterior Deltoid between the two sessions was found statistically significant ( 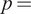 .0391, and 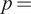 .0078, respectively).

**Figure 6.**
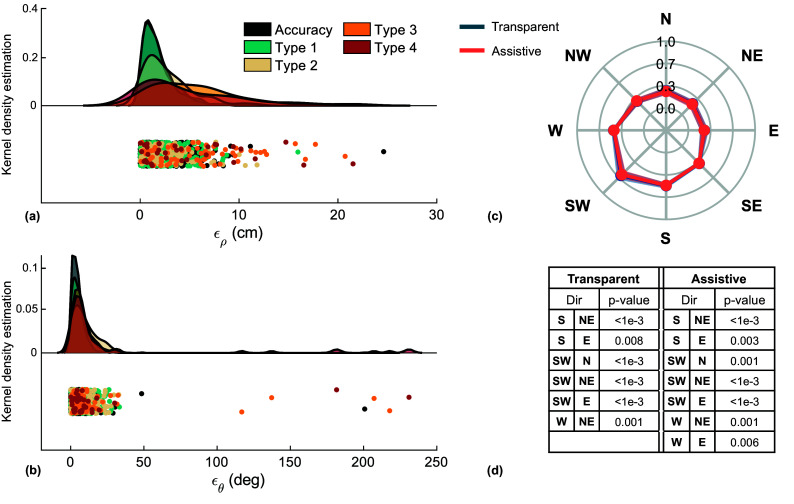
Information derived from NESM- 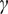 kinematics measurements. (a, b) Raincloud plots of the errors 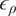 and 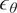 , showing discrepancies in terms of hand advancement and angle, between the final positions of the assisted movements and the targets, derived from transparent session. (c) Spider plot of the mean 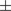 standard deviation manipulability coefficients aggregated on subjects, for the transparent and assisted movements. (d) Direction-dependent statistically significant differences in the manipulability index (only differences with 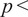 .01 are shown to improve readability).

Figure 6(a, b) presents raincloud plots (Allen et al. 2019) of the errors 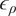 and 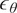 , showing the PDF of the indicators, grouped by error types, as well as the different observations aggregated. Accurate estimates and type 1 errors showed the same median 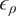 (1.42 cm). Movements causing errors of type 3 and 4 showed the highest discrepancies in terms of hand advancement, showing median 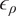 of 4.91 cm and 3.07 cm, respectively. Consistently, the lowest angular errors occurred for accurate and type 1 movements showing a median 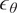 of 3.94 deg and 5.28 deg, respectively. Figure 6(c) shows the distribution of the manipulability coefficients aggregated on participants. On average, the SW direction resulted the one with the highest manipulability both in transparent and assistive sessions (0.62 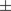 0.08 transparent, 0.61 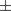 0.07 assistive). Analogously, the NE direction resulted the one with the lowest manipulability in both sessions (0.23 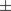 0.06 transparent, 0.22 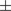 0.06 assistive). A bidirectional Wilcoxon test did not show any significant difference in manipulability indices between transparent and assistive sessions (minimum 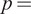 .3125). On the other hand, several direction-dependent significant differences in the manipulability indices were found by the Friedman’s test, when analyzing the movements of the two sessions separately (Figure 6(d)). The strongest differences were found between the SW and NE directions, in both transparent and assistive sessions ( 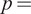 1e−7 and 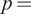 3e−6, respectively).

**Figure 7.**
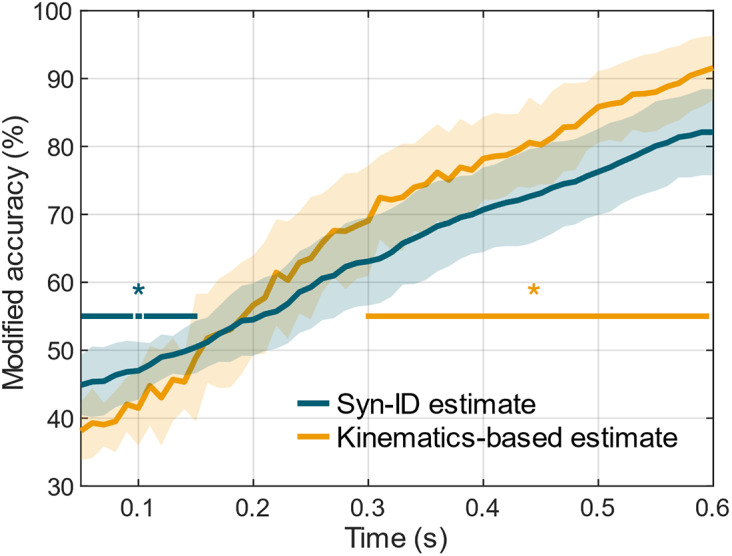
Pattern of the *modified accuracy* (sum of the accuracy and of the errors of type 1) over the accumulation time 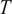 for the proposed muscle synergies-based algorithm and the benchmark algorithm based on kinematics. Horizontal lines with a star highlight the portions of the curves that show significant differences (* 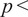 .05).

Finally, Figure 7 shows the evolution over time of the modified accuracy for the Syn-ID and the kinematics-based algorithms, aggregated over subjects. These signals were computed from 0.05 s after the detected EMG onset and were averaged across the subjects. For both signals, the modified accuracy increased over time, reaching a maximum of 82.11 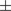 6.34% for the Syn-ID algorithm and 91.58 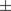 4.76% for kinematics-based benchmark at 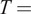 0.6 s. The statistical analyses showed that the Syn-ID algorithm performed significantly better than the benchmark immediately after the onset detection (for 0.14 s; minimum–maximum 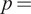 .012–.027, nonsignificant results observed at 0.09 and 0.11 s). On the other hand, the kinematics-based algorithm performed statistically better from 0.29 s after the EMG onset up to the end of the inspected window (minimum–maximum 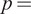 .004–.039).

## 4. Discussions

The choice of inferring the user movement intentions using features derived from muscle synergies is rooted on neuroscientific principles of motor control (d’Avella et al. 2003). Incorporating concepts retrieved by physiological process into the control algorithms of exoskeletons could enhance the interaction between the robot and the user, creating a cognitive interface that is specifically tailored to user’s needs and residual functionalities (Catalán et al. 2023; Crea et al. 2018; Lanotte et al. 2021; Penna et al. 2022, 2023; Ronsse et al. 2011). Subject-specific customization of the exoskeleton behaviour could be particularly effective in poststroke rehabilitation, during which it is important to accommodate the subject’s residual movements (Balasubramanian et al. 2018). The results of this study highlight the feasibility of this approach and open new perspectives in poststroke upper-limb rehabilitation scenarios.

First, the accuracy in the classification of the movement direction based on muscle synergies (48.7%) was notably higher than the chance level adjusted for the number of trials per directions (18.1%, with 95% confidence; Müller-Putz et al. 2008). This result is particularly remarkable considering that the eight movement targets were relatively close to each other in the space and muscle activations (and muscle synergies) were very similar when considering adjacent directions. In line with this, the analysis of error types underlined the prevalence of type 1 errors compared to other error types, showing that most of the confusion of the estimate algorithm resulted along directions adjacent to the actual one. Therefore, the modified accuracy may be considered as the accuracy of the algorithm in detecting movement direction when it is acceptable to identify a more generic direction, namely type 1 errors are acceptable to achieve effective assistance control. In this case, the accuracy reached 82% after 0.6 s from the iEMG onset. The proposed algorithm has comparable or ameliorative results in terms of overall accuracy to state-of-the-art works exploiting EMG-based features to estimate the reaching movement directions. (Irastorza-Landa et al. 2017) obtained an accuracy of 78% using support vector machines to classify the directions of four forward and four backward reaching movements (chance level 25%), using time windows of 1 s for features extraction. (Novak et al. 2013) reached 90% of accuracy at the 75% of movement execution using linear discriminant analysis to classify reaching movements toward three objects (chance level 33%).

In addition to estimate performance, this study assessed the algorithm capability of providing a synchronous and repeatable assistance to the movements of the user through a shoulder-elbow exoskeleton. Overall, the assistive mode allowed participants to move toward the desired target and reach the final position with small errors in terms of hand advancement and angle. Indeed, highest median 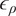 was 4.91 cm, which was notably lower than the radius of the circumference on which the targets were placed (25 cm). Analogously, the highest median angular error was 7.40 deg, which was lower than the angular shift between different targets (45 deg). 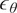 and 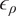 errors were particularly small (median of 5.28 deg, 1.42 cm, respectively) when the classification algorithm classified the direction of movement correctly or with type 1 error. Notably, the median 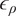 was the same for accurate movements and type 1 error ones.

The effects of the assistance on the user were assessed through EMG activations. Although four out of six muscles showed a reduction of the iEMG values between the transparent and the assistive sessions, only Triceps Brachii and Posterior Deltoid, that is, the extensor muscles of forearm and shoulder, showed statistically significant reductions. This result could be correlated with the values of the manipulability coefficients ( 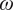 ) of the NESM- 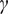 exoskeleton. Indeed, the values of 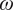 for the north directions (i.e., N, NW, and NE) were significantly lower than the ones for the south ones (i.e., S, SW, and SE). A lower value of the manipulability index results in greater angular displacements in the joint space for an equal displacement of the end effector in the Cartesian space, that is, hand trajectories, which is where the movement planning occurs. This could have caused unexpected muscle contractions that influenced the EMG values of movements toward north directions. For similar reasons, the lower values of 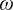 for the north directions could have hindered the classification performance, as reflected by poorer accuracies for N and NE directions (up to 41%). Analogously, the SW and W directions, which have among the highest manipulability coefficients, were also the best estimated directions. The S direction represented an exception, since it was the second highest 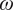 value, but the overall worst estimated direction. Since most of the estimate errors were toward SE (28% of instances), we hypothesized that these were caused by the similar muscle activation patterns of the movements toward these directions, as shown in Figures 3 and 5.

Finally, the performance of the muscle synergies-based algorithm was assessed with respect to a kinematics-based benchmark algorithm, implemented offline. This analysis showed, on average, significant better performance of the synergies-based approach immediately after the EMG onset (up to 0.14 s); subsequently, it followed a phase (from 0.15 to 0.29 s) where the performance of the two algorithms was analogous and no statistically significant differences were observed. The highest performance of the Syn-ID algorithm in these two subphases can be attributed to the electromechanical delay, that is, the time delay that occurs between the EMG and the kinematic onset. From the literature, this value is typically between 0.04 and 0.07 s for forearm free flexion/extension movements without constraints (Cavanagh and Komi 1979); however, it is conceivable that the mechanical coupling of the user with the exoskeleton required higher levels of EMG activations, and thus longer times, to initiate the movement (Irastorza-Landa et al. 2017). Therefore, it is probable that in the initial time instants of the reaching task, the movement intentions of the subjects were not reflected to any detectable kinematic activity, leading to instabilities of the kinematic classification. Conversely, the information carried by the EMG synergies was significantly higher to infer the intended movement. This consideration is emphasized by the fact that, after the electromechanical delay, the kinematics-based algorithm performed better than the synergies-based one, showing statistically significant results until the limit observation time (from 0.3 to 0.6 s). However, considering the implemented assistive strategy, it is crucial to evaluate the potential impact of assistive torques on the kinematic activity of participants, which served as inputs for the benchmark analysis. Despite this potential influence, results suggest that the assistive torques did not disrupt the participants’ voluntary movement patterns. First, as discussed above, the errors in terms of hand advancement and angle in the polar plane were notably lower than the workspace geometries, with only eight movements showing 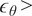 45 deg (0.65% of the assisted movements), suggesting that the assistive torques, despite sporadic severe errors, allowed the participants to complete their voluntary movements. Second, the absence of statistically significant increases in the muscle activities among the two sessions suggests that the participants’ movements were not constrained by the assistance. Finally, the absence of any significant difference in manipulability indices between transparent and assistive sessions (minimum 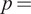 .3125) underlines the coherence of kinematic patterns among the sessions. In this regard, it is noteworthy that the assistance was not tuned with the aim of implementing a user passive mobilization. Rather, the focus was placed on facilitating the subjects’ movements.

This study demonstrated that the observation of EMG-derived features could be useful to provide an initial assistance profile which is sufficiently accurate (50% after ∼ 0.15 s from EMG onset). Nevertheless, the adoption of the proposed approach in rehabilitation scenarios must be first assessed in terms of effectiveness and robustness considering the severely compromised EMG activations typical of poststroke individuals. Several studies reported altered coupling of the shoulder–elbow muscles (Roh et al. 2013) and abnormal contribution of the Deltoid heads in synergies (Tropea et al. 2013), following a stroke. However, there is evidence that supports the employment of muscle synergies in poststroke rehabilitative practice. First of all, studies have shown that four synergies are sufficient to capture the variability in altered EMG activations of poststroke individuals (Israely et al. 2018; Tropea et al. 2013). Second, it was demonstrated that muscle synergies activation coefficients of poststroke individuals exhibit distinctive patterns during three-dimensional reaching tasks toward different targets (Israely et al. 2018). Finally, (Pan et al. 2018) observed consistent activation coefficients between poststroke individuals and a control group of healthy participants during reaching tasks. These results pave the way for the application of a classification logic using muscle synergies-based features in some poststroke populations. The Syn-ID algorithm could be effective for the training of gross upper arm motor functions (e.g., reaching) in patients with limited movement capabilities, who can express muscle activity, but need an amplification to drive the upper-limb toward the desired direction.

The main limitation of the study lies in the assistive strategy, which currently does not include countermeasures in case of misdetections. Indeed, when error of type 3 or 4 occurred, the exoskeleton delivered an assistance that was contrary to the participant’s intended movement. Although the occurrence of these errors was low (8.83% of the total) and healthy participants were still able to execute the desired movements (median 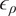 of 2.05 cm and median 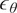 of 6.36 deg), this kind of behavior is not desirable in a rehabilitative scenario, where the participants may not be able to contrast the action of the exoskeleton. Our choice of the assistive strategy in this study was driven by the main objective of verifying the performance of the real-time IDA even when assistance is delivered in the initial instants of the movement. In order to mitigate potential safety concerns in rehabilitation scenarios, the assistive strategy could implement of a veto function, that is, a mechanism that allows the participant to voluntarily interrupt the assistance in presence of unintended behaviors of the system (Clausen et al. 2017; Crea et al. 2018). Alternatively, considering the target end users (i.e., people who can initiate the movement, but who need the assistance to accomplish the task), we could also envision a modification of the assistive strategy, in which the movement is initially performed in transparent mode, and the assistance is enabled only when the estimation has reached a certain cumulative probability level (Tortora et al. 2019).

The current algorithm structure could be improved investigating sensor fusion and sensor integration strategies, mixing the contributions of kinematics and electromyographic information for intention decoding purposes. Indeed, following the electromechanical delay, the kinematic information could be then used to correct the EMG estimate, to further increase the overall accuracy of the intention estimate (Novak et al. 2013). Moreover, classifiers different from GMMs will be tested aiming to improve the estimate accuracy. Additionally, the assistive strategy could be modified to include also compensation for the user’s arm gravitational torques, which could be beneficial for the training of poststroke individuals (Prange et al. 2009). Finally, the robustness of the algorithm will be investigated in subject independent scenarios, aiming at increasing the generalizability of the approach.

## Supporting information

Penna et al. supplementary material Penna et al. supplementary material

## Data Availability

The datasets generated during and/or analyzed during the current study are available from the corresponding author on reasonable request.

## Supplementary material

The supplementary material for this article can be found at http://doi.org/10.1017/wtc.2024.16.
